# 基于巯基化衍生的气相色谱-质谱法测定有机相及水相中氯化氰

**DOI:** 10.3724/SP.J.1123.2020.12004

**Published:** 2021-08-08

**Authors:** Xiaosen LI, Jina WU, Junmei XIA, Ling YUAN, Yang YANG

**Affiliations:** 1.国民核生化灾害防护国家重点实验室, 北京 102205; 1. State Key Laboratory of NBC Protection for Civilian, Beijing 102205, China; 2.防化研究院分析化学实验室, 北京 102205; 2. Laboratory of Analytical Chemistry, Research Institute of Chemical Defence, Beijing 102205, China

**Keywords:** 气相色谱-质谱, 顶空-固相微萃取, 巯基化衍生, 氯化氰, gas chromatography-mass spectrometry (GC-MS), headspace solid phase microextraction (HS-SPME), thiol derivatization, cyanogen chloride (CICN)

## Abstract

氯化氰(ClCN)是常用的化工中间体,也是《禁止化学武器公约》附表颁布的化学毒剂之一。采用传统的比色法或气相色谱法对ClCN进行分析时,稳定性差且检出限高。研究建立了有机相及水相中ClCN的巯基化衍生过程及气相色谱-质谱(GC-MS)的检测方法。经比较后选择1-丁基硫醇作为衍生试剂,有机相中ClCN的衍生条件为衍生温度40 ℃,体系pH=9,反应时间10 min,反应结束后直接进行GC-MS分析。水相中ClCN的衍生条件与有机相相同,衍生完毕后进行顶空-固相微萃取(HS-SPME)。实验考察了萃取温度对萃取吸附效果的影响,确定最佳萃取温度为55 ℃。通过GC-EI/MS确认ClCN的巯基化衍生产物为硫氰酸丁酯,并对质谱图中主要的离子碎片进行结构确认。采用气相色谱-串联质谱法(GC-MS/MS)对硫氰酸丁酯裂解规律进行了分析。采用GC-MS/SIM对有机相及水相中的ClCN进行分析。方法学考察结果表明,ClCN在有机相(20~2000 μg/L)及水相(20~1200 μg/L)中相应的范围内线性关系良好(相关系数(*R* ^2^)>0.99);在3个添加水平下有机相中CICN的回收率为87.3%~98.8%,不同的水相基质中CICN的回收率为97.6%~102.2%, RSD分别为2.1%~4.7%和2.8%~4.2%,衍生过程具有良好的专属性。采用禁止化学武器公约组织(OPCW)的水平考试空白有机样品(样品基质为正己烷)对研究方法进行验证,该方法能够成功检出目标物。该研究建立的巯基化衍生-气相色谱-质谱法灵敏度高,精密度好,能够为环境中ClCN的定性定量分析提供技术支持。

## Abstract (en)

Cyanogen chloride (ClCN) has been widely used in industrial production. ClCN is also listed in the Schedule of the Chemical Weapons Convention (CWC). The use of traditional colorimetric analysis or gas chromatography for the detection of ClCN has been characterized by low efficiency and poor sensitivity. In this study, a method was established for the qualitative analysis and quantitative detection of ClCN in organic and water matrices by gas chromatography-mass spectrometry (GC-MS) based on thiol derivatization. 1-Butylthiol was selected as the optimal derivatization reagent. The optimal temperature for thiol derivatization in the organic matrices was 40 ℃ and the reaction time was 10 min. The pH for derivatization was approximately 9. The ClCN in the organic matrices was directly analyzed by GC-MS after derivatization. The conditions of ClCN derivatization in the water matrices were the same as those in the organic matrices. After the derivatization of ClCN, headspace-solid phase microextraction (HS-SPME) was employed during sample preparation for water matrices. Different temperatures for HS-SPME were explored, and the optimal temperature was found to be 55 ℃. The product of thiol derivatization was confirmed as butyl thiocyanate. The main fragmentation patterns and mass spectrometric cleavage pathway were investigated by GC-MS/MS. The quantitative determination of ClCN in organic and water matrices was conducted via the internal standard and external standard methods, respectively. ClCN showed good linearity in the corresponding ranges in the organic and water matrices. The correlation coefficients for both matrices were greater than 0.99. The linearities of ClCN in the organic and water matrices were in the range of 20-2000 μg/L and 20-1200 μg/L, respectively. An organic sample and water samples from different substrates were selected to verify the accuracy and precision of the method at three spiked levels. The average spiked recoveries of ClCN in the organic sample and water samples were 87.3%-98.8% and 97.6%-102.2%, respectively. The corresponding relative standard deviations (RSDs, *n*=6) were 2.1%-4.7% and 2.8%-4.2%. The derivatization method established in this study showed good reaction specificity. The method was successfully applied in the analysis of samples obtained from the Organisation for the Prohibition of Chemical Weapons (OPCW). The method established in this study for the detection of ClCN showed high sensitivity and precision, and could aid in the analysis and detection of ClCN in the environment.

氰化物是一种常用的工业原料,因其对人体的高毒性和对环境的高风险性,而成为毒剂分析领域的研究热点^[1,2,3,4]^。氯化氰(ClCN)是一种能够引起全身中毒的剧毒气体,属于《禁止化学武器公约》附表中颁布的化学战剂之一^[5]^。我国2001年即建立了水中ClCN的标准检测方法,并制定了相应的含量标准,要求自来水中ClCN含量必须≤70 μg/L^[6]^。水中ClCN的分析主要采用分光光度法,检测机理为氰根与巴比妥酸进行显色反应,然后测定反应物的吸收波长^[7,8,9,10]^。该方法的缺陷在于分析稳定性较差,采样和测定必须在1 d内完成^[11]^,且无法实现ClCN的直接测量分析。

目前国内外已有采用气相色谱法(GC)检测ClCN的研究^[12,13,14,15,16]^,但是GC仅依靠保留时间实现化合物的区分,在样本杂质干扰较大或目标物标准品难以获得的情况下,无法对目标物进行准确定性及定量。采用色谱-质谱技术,通过分析目标物特定的保留时间及质谱碎片,能够实现化合物的准确定性分析及定量检测。但ClCN的相对分子质量小,沸点低(12.6 ℃),且极性较强,当采用GC-MS分析时,ClCN的色谱出峰情况较差,检测灵敏度低^[17,18]^。Nagashima等^[19]^使用便携式气相色谱-质谱仪对ClCN直接分析,检出限为10 mg/m^3^。Yang等^[20]^尝试对ClCN直接进行GC-MS分析,检出限高达1.2~1.7 mg/L,无法满足痕量检测的要求。同时,ClCN在常温下挥发性高且毒性大,因此直接对样品中未知浓度的ClCN进行分析具有一定安全隐患。采用液相色谱-质谱法(LC-MS)对ClCN进行分析时,ClCN会在液相色谱的流动相中发生水解,影响定量分析结果^[21]^。

通过衍生化手段将低沸点、极性强且色谱分离效果差的氯化氰衍生为极性较弱且稳定性高的衍生化产物,进一步通过GC-MS对氯化氰的衍生产物进行分析,能够获得良好的色谱分离效果以及高检测灵敏度,满足氯化氰的痕量检测要求。目前针对ClCN衍生方法的相关研究报道较少。巯基化衍生是衍生试剂分子中活泼的巯基(-SH)与目标物直接进行反应的过程。已有研究报道^[22]^将巯基化衍生技术应用于化学毒剂(如糜烂性毒剂路易氏剂)等的分析中。与传统的硅烷化衍生^[23]^等柱前衍生方法相比,巯基化衍生过程通常无需加热,仅在室温下反应数分钟即可完成,大大缩短了衍生时间。本研究通过巯基化衍生的方法,采用1-丁基硫醇作为衍生化试剂,将ClCN衍生为硫氰酸酯,并进行气相色谱-质谱的定性分析及定量检测。以异丙基二硫醚作为内标物,采用内标法对有机相中的ClCN衍生产物进行分析;应用顶空-固相微萃取(HS-SPME)技术对水中ClCN的巯基化衍生产物进行富集,并采用外标法对水相中的ClCN衍生产物进行分析。本研究建立的样品制备及分析方法灵敏度高,选择性好,样品分析结果稳定,适合用于环境中ClCN的分析。

## 1 实验部分

### 1.1 仪器与试剂

7890A/5975C气相色谱-质谱联用仪(美国Agilent公司); Trace GC Ultra/TSQ Quantum XLS气相色谱-串联质谱仪(美国Thermo公司)。

甲苯、二氯甲烷、氯仿购自北京百灵威科技有限公司(色谱纯,纯度≥99%);乙酸、氯化钠、硫酸钠、硼酸钠、氢氧化钠购自国药集团化学试剂北京有限公司(分析纯,纯度≥97%);去离子水购自屈臣氏;1-丁基硫醇、1-丙基硫醇、1-乙基硫醇、3,4-二巯基甲苯、异丙基二硫醚、三乙胺购自美国Sigma公司(分析纯,纯度≥97%)。聚二甲基硅氧烷(PDMS)固相微萃取纤维购自美国Supelco公司(膜厚度100 μm)。ClCN由实验室按照文献^[24]^路径合成,纯度≥99%。河水采自北京市昌平区阳坊镇京密引水渠;自来水由北京市供应;饮用水为娃哈哈瓶装矿泉水。

### 1.2 样品制备与衍生条件

准确称取两份2.0 mg ClCN样品,置于4.0 mL螺纹玻璃瓶中,各加入2.0 mL甲苯及去离子水,配制1.0 mg/mL ClCN/甲苯和ClCN/去离子水溶液,并充分振荡溶解。

移取500 μL ClCN/甲苯和ClCN/去离子水溶液,置于1.5 mL螺纹玻璃瓶中,分别加入5 μL 1-丁基硫醇。根据反应体系的不同,加入5 μL三乙胺(甲苯溶液)或5 μL 1.0 mmol/L氢氧化钠溶液(水溶液)调节反应体系的pH值至9,将螺纹玻璃瓶盖紧后混合均匀,于40 ℃加热条件下衍生反应10 min,反应结束后自然冷却至室温。

衍生反应方程式如下:


(1) ClCN+CH_3_CH_2_CH_2_CH_2_-SH→CH_3_CH_2_CH_2_CH_2_S-CN+HCl

### 1.3 样品前处理

衍生完毕后,在ClCN/甲苯衍生样品中加入5 μL含10 mg/L异丙基二硫醚的甲苯溶液,振荡混合均匀后取1.0 μL进行GC-MS分析。

对于ClCN/去离子水衍生样品,衍生完毕后,采用固相微萃取的方式进行样品前处理。将PDMS固相微萃取纤维通过瓶盖隔垫插入螺纹玻璃瓶中,萃取纤维距离液面约5 mm。衍生反应体系在55 ℃加热条件下完成顶空-固相微萃取,萃取时间5 min。萃取完毕后拔出固相微萃取纤维,直接插入气相色谱-质谱联用仪的进样口脱附2 min。

### 1.4 仪器条件

1.4.1 色谱条件

色谱柱:Agilent DB-5MS柱(30 m×0.25 mm×0.25 μm);载气:氦气;恒流模式,流速:1.0 mL/min;进样口温度:250 ℃;不分流进样:0.7 min;溶剂延迟:3 min;柱升温程序:起始温度40 ℃,保持1 min,以10 ℃/min的升温速率升至280 ℃,保持5 min。

1.4.2 质谱条件

离子源EI源;离子源温度250 ℃;四极杆温度230 ℃;传输线温度250 ℃;电子能量70 eV。全扫描分析时,扫描范围及速率:*m/z* 29~550,0.5 s;子离子扫描时,分别选择*m/z* 115(碰撞能量10 eV)及*m/z* 57(碰撞能量5、10及20 eV)作为母离子;选择离子监测模式(SIM)时,ClCN衍生产物定性和定量离子的*m/z*分别为115和41,内标物定性和定量离子的*m/z*分别为150和43。

### 1.5 安全措施

所有涉及ClCN的操作均需要佩戴相应的防护器材,并在通风良好的状况下进行。配制饱和的氢氧化钠水溶液作为洗消剂,各种直接接触ClCN的实验器材在使用后立即洗消(操作人员经过专业培训,配备相应急救措施)。

## 2 结果与讨论

本研究首先考察直接进样条件下氯化氰的色谱行为及质谱出峰情况,接着对ClCN进行巯基化衍生,并进行正交试验,以获得最优反应条件。通过GC-MS/MS对ClCN的衍生产物进行质谱裂解规律解析,最终建立GC-MS/SIM定量分析方法并进行方法学考察。

### 2.1 直接分析ClCN的色谱行为考察

取1 μL 10 mg/LClCN/甲苯标准工作溶液,按1.3节所述进行全扫描分析。如图1所示,目标物的出峰时间非常靠前(*t*_R_为1.62 min)且色谱峰出现严重拖尾,难以对ClCN进行准确定量。这可能是因为ClCN相对分子质量较小,沸点低且极性强,这也是目前对ClCN进行色谱分析的难点所在。因此本研究采用衍生的方式,将ClCN衍生为沸点较高、极性较弱的衍生产物,以获得更好的色谱峰形,并实现ClCN的定量分析。

**图1:**
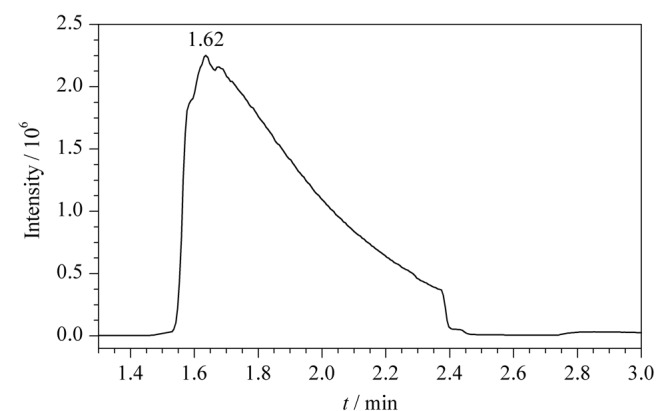
ClCN(10 mg/L)/甲苯标准工作溶液的色谱图

### 2.2 衍生试剂的选择

以甲苯为溶剂分别考察了有机相中4种巯基化衍生试剂(1-乙基硫醇、1-丙基硫醇、1-丁基硫醇和3,4-二巯基甲苯)的衍生效果,实验结果如图2所示。采用1-乙基硫醇和3,4-二巯基甲苯进行衍生后找不到衍生产物的色谱峰。采用1-丁基硫醇衍生后,ClCN衍生产物的峰面积大于1-丙基硫醇。因此本实验采用1-丁基硫醇作为巯基衍生试剂,衍生产物为硫氰酸丁酯,保留时间为7.468 min

**图2:**
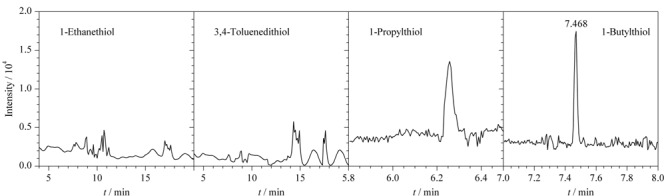
采用不同衍生试剂时ClCN衍生产物的色谱图

### 2.3 衍生条件的优化

分别配制不同有机溶剂的ClCN(1 mg/L)标准工作溶液,采用1-丁基硫醇作为衍生化试剂,设计四因素三水平的正交试验,每组实验平行重复3次,结果见表1。根据显著性分析结果可知,在所选因素水平范围内,影响衍生结果的显著性次序为:体系pH值>衍生温度>衍生时间>溶剂体系,其中只有体系pH值对实验结果有显著性影响。当反应体系的pH=9时,ClCN衍生产物的色谱峰面积要明显高于中性及酸性体系中衍生产物的峰面积。

**表1:** 正交试验结果

Level	Temperature/ ℃	Time/min	Solvent	pH value
1	30	5	toluene	9 (adjusted by triethylamine)
2	40	10	dichloromethane	no addition
3	50	20	chloroform	5 (adjusted by acetic acid)
Mean square	5.12×10^8^	2.13×10^8^	4.32×10^7^	1.24×10^11^
*F*	2.48	1.08	0.21	874.2
Significance	no influence	no influence	no influence	highly significant

根据衍生反应方程式可知,在体系中加入缚酸剂能够有效促进衍生反应向正反应方向进行。考虑到时间成本以及合适的温度,最优衍生条件为:反应温度40 ℃,衍生时间10 min,反应体系pH=9,有机相和水相分别采用三乙胺及氢氧化钠作为缚酸剂。

### 2.4 顶空-固相微萃取条件优化

考察不同顶空-固相微萃取温度对实验结果的影响。在室温(25 ℃)下对含500 μg/L ClCN/去离子水进行巯基化衍生后,在不同的温度(25、35、45、55、65、75、85 ℃)下进行顶空-固相微萃取吸附。每个温度下进行3次重复试验,比较不同顶空温度下ClCN衍生产物的峰面积(见图3)。

**图3:**
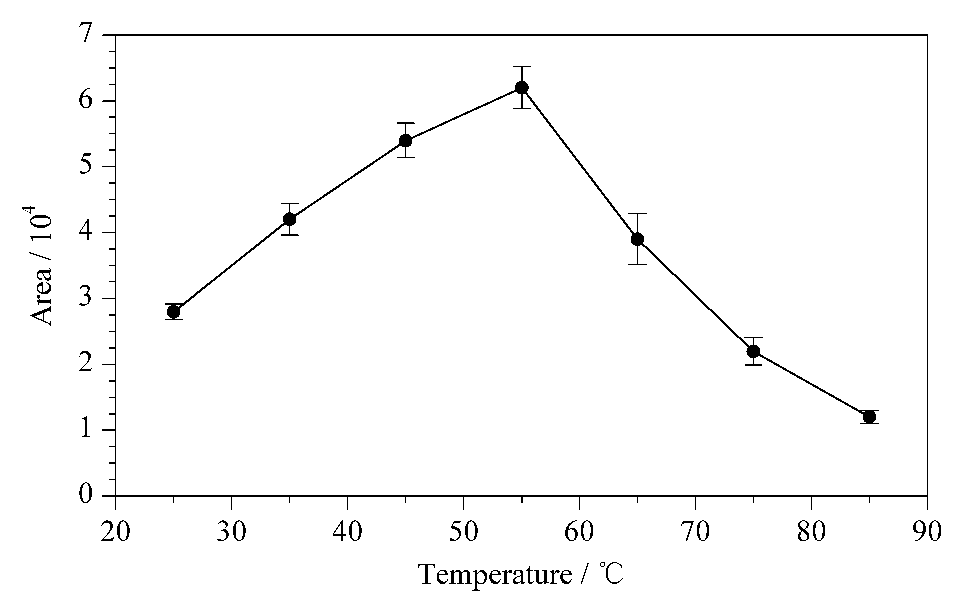
不同顶空-固相微萃取温度对ClCN衍生产物峰面积的影响(*n*=3)

结果表明,当顶空温度为25~55 ℃时,目标物随着顶空温度的上升,峰强度增加。当顶空温度超过55 ℃时,目标物峰面积减小,表明顶空温度的进一步升高对固相微萃取过程不利。可能原因是升温有助于目标物的挥发,而温度过高会导致固相微萃取纤维的解吸,不利于目标物在萃取纤维上的富集。因此本实验选择55 ℃作为顶空-固相微萃取温度。

500 μL 10 mg/L ClCN/去离子水溶液采用固相微萃取进行样品制备及仪器分析时,目标化合物的保留时间与有机相时不同(见图4),为6.844 min,原因在于萃取纤维的吸附/解吸速率与直接进样的气化速度不同。

**图4:**
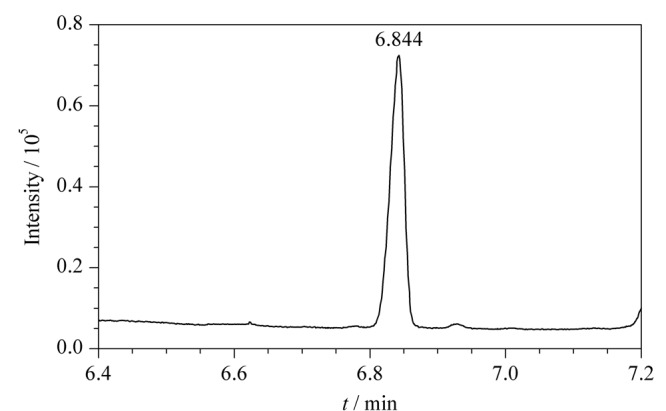
水样基质中10 mg/L ClCN衍生产物的色谱图

### 2.5 衍生产物的鉴定及质谱裂解规律的分析

根据ClCN巯基化衍生产物的反应式和GC-EI/MS质谱图,可知衍生产物为硫氰酸丁酯,主要质谱碎片离子的*m/z*为29、41、57、115等。

进一步对硫氰酸丁酯的碎片离子峰进行归属并推断其质谱裂解规律:*m/z*为115的碎片离子峰代表衍生产物硫氰酸丁酯的分子离子峰,*m/z*为57和29的碎片离子分别代表硫氰酸丁酯经电子轰击电离后产生的丁烷基和乙烷基碎片离子。但是质谱图中*m/z*为41的碎片离子无法直接归属。

采用GC-MS/MS子离子扫描的方式进一步对分子碎片进行碰撞解析。首先选择*m/z*为115的质谱碎片作为母离子,碰撞能量设为10 eV。在子离子的质谱图中发现了*m/z*分别为115、57、41、和29的碎片离子。该结果证明,*m/z*为29、41、57的碎片离子由衍生产物硫氰酸丁酯的分子离子经碰撞裂解后所产生。

进一步选择*m/z*为57的碎片离子作为母离子,在不同的碰撞能量(5、10及20 eV)下进行裂解。如图5所示,不同的碰撞能量下均可发现*m/z*为41的质谱碎片。该结果证明,*m/z*为41的质谱碎片可由*m/z*为57的质谱碎片进一步碰撞裂解得到,而并非来自硫氰根(-SCN)。这与烷烃质谱的裂解规律相符,*m/z*为41的碎片离子来自于碳正离子的裂解,符合偶电子规律。同时根据质谱丰度可知5 eV的碰撞能量最佳。

**图5:**
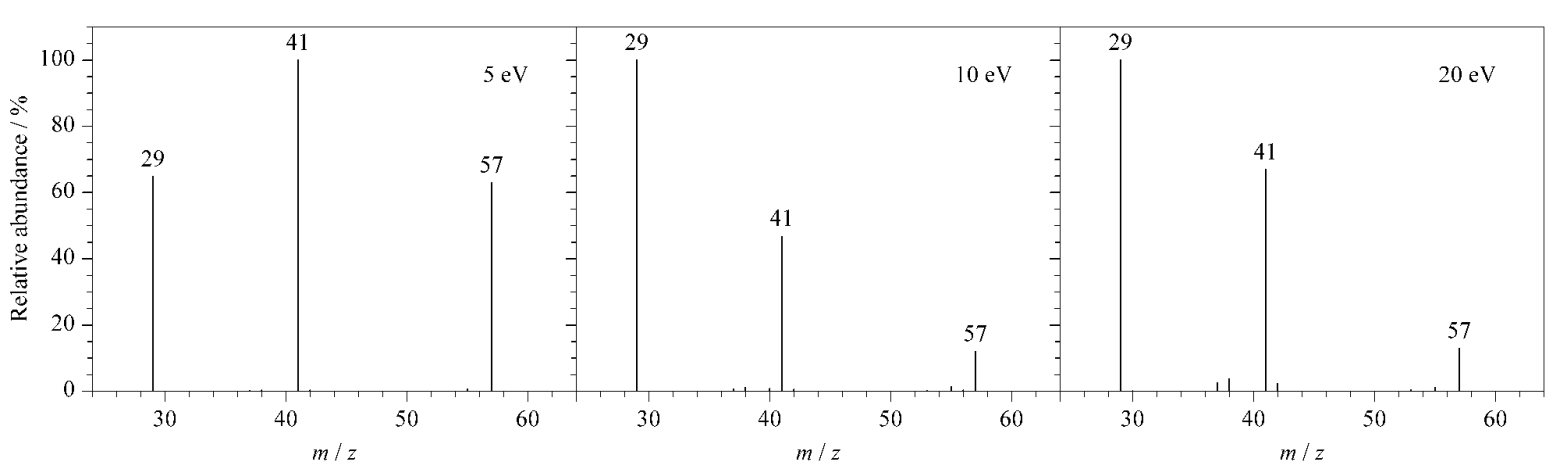
不同碰撞能量下*m/z* 57的碎片离子作为母离子时的质谱图

根据GC-MS/MS结果,对所有的碎片离子进行归属并推断硫氰酸丁酯在电子轰击电离下的裂解途径。如图6所示,推断*m/z*=41的离子碎片为烯丙基碳正离子结构。

**图6:**
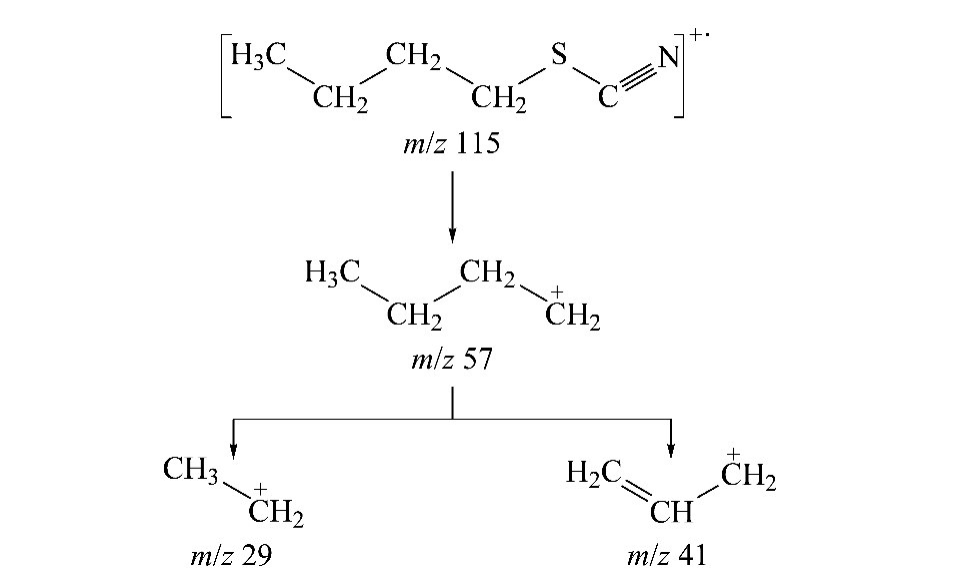
硫氰酸丁酯的质谱裂解规律

通过对ClCN衍生产物质谱图中主要碎片离子的分析,能够推断化合物在电子轰击条件下的裂解途径,有助于理解并阐明主要质谱碎片之间的相互关系,并为未来建立痕量ClCN的GC-MS/SRM(选择反应监测扫描)方法提供技术储备。

### 2.6 定量方法的选择

采用内标法进行定量测定能够避免实验前处理操作中目标成分丢失造成的定量不准确,还能有效提高检测灵敏度并降低样品制备过程中的干扰。本研究在实验条件允许的情况下,采用内标法对有机相中ClCN进行定量分析,以获得更好的定量准确度。

异丙基二硫醚具有较好的化学稳定性,方便保存,且高纯度标准品易于购买。同时异丙基二硫醚与衍生产物均含S元素,化学性质较为类似。在本研究中的色谱及质谱条件下,异丙基二硫醚的保留时间(8.050 min)与目标物接近(7.468 min),且色谱峰能够完全分离。因此,选择异丙基二硫醚作为内标物进行有机相中ClCN的定量分析。

水相中采用了顶空-固相微萃取的方式对ClCN衍生产物进行富集,如果在样品中加入内标物,在顶空加热过程中内标物会与ClCN衍生产物在固相微萃取纤维上发生竞争性吸附,从而影响检测灵敏度以及实验结果的精密度。因此水相中的ClCN采用外标法定量。

### 2.7 方法学考察

2.7.1 线性范围,检出限和定量限

分别配制10~2000 μg/L ClCN/甲苯标准工作溶液及10~1200 μg/L ClCN/去离子水标准工作溶液,依次根据1.2节和1.3节进行制备和测定。

在有机相中选择异丙基二硫醚为内标物,以ClCN的质量浓度为横坐标(*x*, μg/L)、ClCN衍生产物与内标物峰面积之比为纵坐标(*y*_1_)进行线性拟合。对于水相中ClCN的测定,以ClCN的质量浓度为横坐标(*x*, μg/L), ClCN衍生产物峰面积为纵坐标(*y*_2_)进行线性拟合。

以响应显著高于溶剂空白且信噪比为3对应的浓度作为方法的检出限(LOD),以线性范围最低点的浓度作为方法的定量限(LOQ)。有机相及水相中ClCN的线性方程、检出限和定量限结果见表2。

**表2:** 不同基质中ClCN的线性范围、线性方程、相关系数(*R*^2^)、检出限及定量限

Matrix	Linear range/ (μg/L)	Linear equation	*R* ^2^	LOD/ (μg/L)	LOQ/ (μg/L)
Organic	20-2000	*y*_1_=2.4781*x*-1.0415	0.9972	15	20
Water	20-1200	*y*_2_=2.5893*x*+0.8397	0.9902	17	20

*y*_1_: peak area ratio of derivatives of ClCN to IS; *y*_2_: peak area; *x*: mass concentration, μg/L.

2.7.2 加标回收率与精密度

以甲苯及不同来源的水样(去离子水、河水、自来水、饮用水)作为样品基质,分别对制3个水平的ClCN基质溶液进行加标回收试验,每个水平平行测定6次(见表3)。结果表明,在甲苯中ClCN的加标回收率为87.3%~98.8%, RSD为2.1%~4.7%;在不同的水样基质样本中ClCN的加标回收率为97.6%~102.2%, RSD为2.8%~4.2%。表明本方法的稳定性和重复性均较好。

**表3:** 不同基质中ClCN的加标回收率和相对标准偏差(*n*=6)

Matrix	Spiked level/(μg/L)	Recovery/%	RSD/%
Toluene	40	98.8	4.7
	900	93.4	3.3
	1800	87.3	2.1
Deionized water	100	98.2	3.2
	500	97.6	3.1
	1000	98.9	2.8
River water	100	102.2	3.3
	500	100.4	4.2
	1000	100.2	3.2
Tap water	100	98.5	2.8
	500	98.3	3.4
	1000	99.8	3.5
Drinking water	100	101.3	3.1
	500	100.3	3.2
	1000	101.4	2.9

优良的衍生体系能够对目标化合物进行专一性的反应。考虑到杂质干扰可能对衍生反应造成假阳性结果,选择氯化钠、硫酸钠、硼酸钠作为干扰离子加入ClCN/去离子水标准工作溶液中,配制成空白基质加标样品(干扰离子与ClCN最终浓度均为500 μg/L);以去离子水为基质配制氯化钠、硫酸钠、硼酸钠混合溶液(最终浓度均为500 μg/L),作为基质空白;以去离子水作为溶剂空白。对空白基质加标样品、基质空白及溶剂空白分别进行分析。结果表明,Cl^-^、$SO_{4}^{2-}$、$B_{4}O_{7}^{2-}$对衍生反应无影响,证实巯基衍生过程对于ClCN具有良好的专属性。

### 2.8 实际样品测定

本实验室为禁止化学武器组织(OPCW)指定实验室,参与OPCW第33次水平考试的配样过程。取第33次水平考试的空白有机样品(样品基质为正己烷,样品编号333),加入ClCN标准工作溶液,使其最终质量浓度为1000 μg/L(选择该浓度的原因是OPCW水平考试中规定目标化合物最低添加含量为1000 μg/L)。取该样品1.0 mL,根据已建立的方法测定样品中CNCl的含量,结果为982.3 μg/L,RSD为1.13%(*n*=3)。表明采用本实验建立的方法能够有效测定模拟加标实际样品中CNCI的含量。

## 3 结论

本研究建立了巯基化衍生-气相色谱-质谱测定氯化氰的方法,并对衍生产物的质谱裂解规律进行了探究。针对不同溶剂的样本,分别通过直接衍生及顶空-固相微萃取的方式进行了样品制备过程。本方法灵敏度高,选择性好,能够实现不同基质样本中氯化氰的定性分析及定量测定。
